# Metatranscriptomics reveals metabolic adaptation and induction of virulence factors by *Haemophilus parasuis* during lung infection

**DOI:** 10.1186/s13567-015-0225-9

**Published:** 2015-09-23

**Authors:** Bernardo Bello-Ortí, Kate J. Howell, Alexander W. Tucker, Duncan J. Maskell, Virginia Aragon

**Affiliations:** Centre de Recerca en Sanitat Animal (CReSA), Institut de Recerca i Tecnologia Agroalimentàries (IRTA), Campus de la Universitat Autònoma de Barcelona, Bellaterra, 08193 Cerdanyola del Vallès, Spain; Department of Veterinary Medicine, University of Cambridge, Madingley Road, Cambridge, CB3 0ES UK

## Abstract

*Haemophilus parasuis* is a common inhabitant of the upper respiratory tract of pigs, and the causative agent of Glässer’s disease. This disease is characterized by polyserositis and arthritis, produced by the severe inflammation caused by the systemic spread of the bacterium. After an initial colonization of the upper respiratory tract, *H. parasuis* enters the lung during the early stages of pig infection. In order to study gene expression at this location, we sequenced the ex vivo and in vivo *H. parasuis* Nagasaki transcriptome in the lung using a metatranscriptomic approach. Comparison of gene expression under these conditions with that found in conventional plate culture showed generally reduced expression of genes associated with anabolic and catabolic pathways, coupled with up-regulation of membrane-related genes involved in carbon acquisition, iron binding and pathogenesis. Some of the up-regulated membrane genes, including ABC transporters, virulence-associated autotransporters (*vtaA*s) and several hypothetical proteins, were only present in virulent *H. parasuis* strains, highlighting their significance as markers of disease potential. Finally, the analysis also revealed the presence of numerous antisense transcripts with possible roles in gene regulation. In summary, this data sheds some light on the scarcely studied in vivo transcriptome of *H. parasuis*, revealing nutritional virulence as an adaptive strategy for host survival, besides induction of classical virulence factors.

**Electronic supplementary material:**

The online version of this article (doi:10.1186/s13567-015-0225-9) contains supplementary material, which is available to authorized users.

## Introduction

*Haemophilus parasuis* is the causative agent of Glässer’s disease, an infectious disease of pigs characterised by fibrinous polyserositis. Current strategies for disease control are based on rapid diagnostics, the use of antibiotics and to a lesser extent vaccines [1]. Antibiotics have been extensively used for this purpose, but current recommendations focus on reduction of their use to avoid the emergence of drug resistance [2-4]. Antibodies can control disease [5] in a mechanism that, at least in part, relies on opsonisation, which renders the virulent phagocytosis resistant strains susceptible to killing by alveolar macrophages [6]. Vaccines, as well as probiotics, are candidates to replace antimicrobials as preventive agents [7,8]. Virulence factors, especially those important for the initial stages of infection, are ideal targets for vaccine design in order to block the pathogenesis potential of bacteria. In that regard, some *H. parasuis* virulence factors have been reported in the literature, and were reviewed recently [9,10].

Numerous works have indirectly linked specific *H. parasuis* genes to its pathogenicity, but direct demonstration of their role during infection is still lacking. In addition, these studies have been typically driven by the homology to previously reported virulence factors in other bacterial species from the *Pasteurellaceae* family. Moreover, pathogenic mechanisms, such as immunomodulation or mechanisms for nutrient acquisition during host infection, could be linked to unsuspected virulence factors [11,12]. After intranasal inoculation, virulent *H. parasuis* can be detected in the lung, from where it can spread causing systemic infection, with the consequent severe inflammation [13,14]. In the lung, *H. parasuis* is detected inside macrophages and neutrophils, but also within epithelial cells [14]. Survival of *H. parasuis* in the lung environment seems to be linked to phagocytosis resistance capacity of the strain, but other unknown virulence mechanisms cannot be ruled out [14,15]. To address this issue, in vivo approaches coupled with hypothesis generating strategies, such as high-throughput RNA sequencing (RNA-seq), could add additional insight into *H. parasuis* pathogenic mechanisms. To our knowledge, no studies have been reported regarding transcriptomic analysis of *H. parasuis* during infection. Few papers have been published in the *Pasteurellaceae* family, but only Jorth et al. [16] applied high-resolution transcriptomics [16-19]. To fill this gap in *H. parasuis* infection control, we used a metatranscriptomic approach to study *H. parasuis* pathogenesis in the pig lung. Gene expression profiling, and more recently RNA-seq, has been established as the *de facto* gold standard technique to tackle the survival strategies of numerous bacterial pathogens [20-22]. The specific objective of this work was to study *H. parasuis* gene expression during lung infection, with a special focus on previously reported virulence factors [10]. We found that *H. parasuis* changes its global gene expression during lung infection. A down-regulation of *H. parasuis* metabolism in the lung was accompanied by the induction of the expression of known virulence-factors together with genes of unknown function.

## Materials and methods

### RNA samples and sequencing

The virulent *H. parasuis* Nagasaki strain was chosen for transcriptomic analysis [GenBank: ANKT01000000]. This strain was originally isolated from the meninges of a pig with a systemic infection by *H. parasuis* in Japan. Gene annotations are based on previous analysis [23]. Further pathway inspection was performed with Integrated Microbial Genomes (IMG) [24] and BioCyc [25].

Animal experiments were performed in accordance with the regulations required by the Ethics Commission in Animal Experimentation of the Generalitat de Catalunya (Approved Protocol number 5796).

To examine gene expression during lung infection, ex vivo incubation of the bacteria in porcine lungs was carried out. Nagasaki grown overnight on chocolate agar plates was resuspended in a final volume of 20 mL sterile PBS (aprox. 10^7^ CFU/mL). A healthy 6 week old pig was euthanised and lungs were extracted under sterile conditions and transported to the laboratory, where they were inoculated with the bacterial suspension. After 2 h of incubation at 37 °C with 5% CO_2_, bacteria were recovered from the lung by lavage with 100 mL of cold sterile PBS. Colony counts were performed before and after incubation. Next, the brochoalveolar lavage fluid (BALF) was subjected to differential centrifugation to eliminate pulmonary cells (460 × *g*, 10 min) and recover bacteria in a second centrifugation (3220 × *g*, 20 min). The bacterial pellet was processed for RNA extraction following two previously described hot phenol extractions [26]. The protocol included depletion of host and bacterial rRNA with a Ribo-Zero kit (Epicentre). This bacterial RNA sample is referred to as the ex vivo sample for simplicity through the rest of the text. A sequencing library was generated using an Ion Torrent RNA-Seq v2 kit (Life Technologies) and RNA was sequenced using an Ion Torrent PGM instrument (Life Technologies) with an Ion 316 chip (Life technologies) at the Centre for Research in Agricultural Genomics (CRAG, Campus de Bellaterra-UAB, Spain). RNA from the Nagasaki strain grown overnight on chocolate agar plates was purified and sequenced in the same manner and served as the control. A replicate of the complete experiment was processed in the same manner.

To assess the validity of the ex vivo model, RNA was obtained from Nagasaki recovered from the lungs of a pig after a short in vivo infection. Nagasaki grown overnight at 37 °C and 5% CO_2_ on chocolate agar plates was resuspended in 20 mL of PBS (aprox. 10^7^ CFU/mL). This bacterial suspension was intratracheally inoculated in a 6 week old pig. A second pig was mock inoculated with the same quantity of PBS. After two hours, the two pigs were euthanized and lungs were processed as above to obtain a bacterial pellet. This bacterial sample is referred to as the in vivo sample for simplicity through the rest of the text. The pellets from the low speed centrifugation containing alveolar macrophages were processed to study the expression of surface markers CD163, sialoadhesin (or CD169), SLAI and SLAII by flow cytometry following a previously described protocol [27]. Bacterial RNA was purified as above and libraries were generated using NEBNext® Ultra™ Directional RNA Library Prep Kit for Illumina®. Sequencing in one paired-end Illumina MiSeq run was performed at the Institut de Biotecnologia i Biomedicina (IBB, Campus de Bellaterra-UAB, Spain). RNA from the Nagasaki strain grown overnight on chocolate agar plates was purified and sequenced in the same manner and served as the control. The NEBNext Ultra Directional kit uses the dUTP method to preserve “directionality of the library”.

### Transcriptomic analysis

RNA-seq analysis was based on the *H. parasuis* Nagasaki draft genome. The genome is 2.3 Mb in length and was assembled in 47 scaffolds [23]. The Nagasaki genome encodes 2260 protein-coding genes (with 40 additional putative pseudogenes): 329 annotated as transporters, 754 connected to KEGG pathways and 1418 to KEGG orthology (KO), 1571 are COG annotated and 1918 associated to Pfam. This information is available in IMG.

For both in vivo and ex vivo experiments*,* bioinformatic analysis was performed following the count-based differential expression method [28], with some modifications [26]. The procedure was the same as that used previously; however, BWA aligner v0.7.9a-r786 was used [29] and minimum mapping quality (MAPQ) was increased to 20. SAMStat was used to get additional mapping statistics [30]. Only protein coding genes with one or more counts per million (cpm) in at least one of the samples compared were used. EdgeR tool was chosen for differential gene expression, defining differentially expressed genes at *FDR* < 0.05. EdgeR biological coefficient of variation (BCV) was calculated using the data from the ex vivo experiment, and was also used to analyse the in vivo data. Enrichments were performed using the BLAST2GO built-in tool [31], using *P* < 0.05 as the threshold. Enrichment results were further processed using the REVIGO online tool [32].

In an attempt to detect putative virulence factors, up-regulated genes in the in vivo or ex vivo experiments compared to agar plate growth were further analysed. Transmembrane domains and the presence of signal peptide motifs were predicted using Phobius [33]. In addition, the presence of these membrane-related genes in a database containing 10 virulent and 14 non-virulent isolates of *H. parasuis* was also studied. This database contains some of the *H. parasuis* genomes that were recently published [34] [EMBL:ERS132054- EMBL:ERS132066, EMBL:ERS132069, EMBL:ERS132073, EMBL:ERS132075, EMBL:ERS132076, EMBL:ERS132078- EMBL:ERS132084]. Automated genome annotation was performed by Howell et al. [34]. The predicted proteome of Nagasaki was compared against the database of *H. parasuis* proteins using BLASTP with a cut-off value of E = 1 × 10–5. Proteomes were parsed from GenBank files using a Biopython script. The settings to define homology were the following: identities ≥ 90% and query coverage ≥ 80% per high-scoring segment pair (HSP). All transcriptomic data were deposited in the Gene Expression Omnibus database [GEO: GSE63851].

Non-coding RNA (ncRNA) were also examined. Read mapping to the complement strand were analysed with the same pipeline as the sense reads, including differential expression and enrichment. Antisense RNA (asRNAs) have been reported in the literature overlapping more than one gene, but also partially overlapping the target gene, commonly known as *cis*-encoded sRNA. For simplicity, in this study we took one gene as the unit of analysis, and therefore we did not take into account the possible overlap of an asRNA with more than one gene. Reverse transcription (RT)-PCR was performed to validate four asRNA using SuperScriptTM III Reverse Transcriptase (Life Technologies). Only a forward primer was used in the RT step. Each putative asRNA was validated in plate and in vivo RNA samples, using Nagasaki DNA as a positive control. To ensure DNA absence, samples without prior reverse transcription were also amplified.

### RT-qPCR

To validate in vivo and ex vivo RNA-seq runs, primers were designed for selected genes (listed in Additional file 1). Also, some putative virulence factors were validated in these two samples, as well as in additional in vivo samples from a previous experiment [27]. Briefly, snatch-farrowed colostrum-deprived piglets were intranasally inoculated with 5 × 10^6^ CFU of Nagasaki. After 1, 2 and 3 days post infection (dpi), pigs were euthanized and lungs were recovered. Bronchoalveolar lavage was performed and bacteria were obtained for RNA purification as described above. Samples were retrotranscribed using SuperScript® VILO™ Master Mix (Life Technologies). qPCR were performed in triplicates in the 7500 Fast Real-Time PCR System with SYBR® Green Real-Time PCR Master Mix (Life Technologies). Each sample belonged to one animal at the specific time post infection. Expression of the selected genes was compared to plate culture. The results were analysed using the ΔΔC_t_ method.

## Results

As a facultative anaerobe, *H. parasuis* Nagasaki is expected to have the ability to generate energy via fermentation or respiration. In fact, when metabolic pathways were inspected using IMG and BioCyc databases, genes involved in both aerobic and anaerobic respiration were detected (oxygen and nitrate as terminal electron acceptors), as well as formate and acetate fermentation genes. Sugar transport systems such as ATP-binding cassette (ABC) transport complexes and phosphotransferase systems (PTS) were encoded in its genome, as well as the genes associated to glycolysis, gluconeogenesis, tricarboxylic acid (TCA) cycle and the pentose phosphate pathway. From the anabolic side, pathways for the biosynthesis of amino acids, nucleotides, cofactors, fatty acids as well as heme were present. Natural competence, adherence and secretion capabilities, lipooligosaccharide (LOS) biosynthesis, proteases and iron acquisition completed the repertoire of genes that should give the Nagasaki strain the potential to colonize and survive during host infection.

In order to mine RNA-seq results, a custom GO term database was built to include more functionally annotated genes and therefore be potentially more suitable for pathway mining than the KEGG annotations assigned by IMG. Using this custom database, at least one GO term was assigned to 74% of the 2260 protein coding genes. A good GO level distribution (mean level = 6.8; SD = 2.7) and more than 8000 annotations were used for pathway mining. Since 25% of the genes did not have any associated GO term, they were not taken into account when performing enrichments, and a separate analysis had to be performed to mine possible up-regulated membrane-related genes.

### RNA-seq run statistics

**Figure 1:**
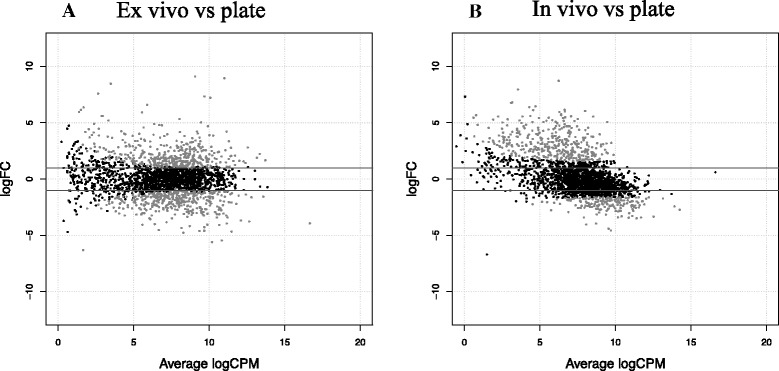
**MA plots generated by EdgeR.** Transcript expression profiles in the two comparisons performed: ex vivo vs plate culture (**A**) and in vivo vs plate culture (**B**). For each gene, log_2_(fold change) between the two conditions is plotted (M, y axis) against the gene’s log_2_(average expression) in the two samples (**A**, x axis). Horizontal lines indicate 2-fold changes. Grey dots highlight the genes at 5% *FDR*.

A range of 2.8-3.2 M and 2.6-3.7 M reads were obtained with Ion Torrent from the ex vivo samples and the corresponding plate samples, respectively. Alignment to the Nagasaki genome was successful for 53-87% of the reads from the ex vivo samples and 61-90% of the reads from the pure plate cultures. To perform differential expression, BCV was calculated and a value of 0.3 was obtained. Differential expression analysis of the ex vivo samples revealed 765 differentially expressed genes (DEG) with statistical significance (*FDR* < 0.05). From these, 393 were up-regulated and 372 down-regulated after 2 h incubation in lung explants (Figure 1A and Additional file 2A). RNA-seq results were validated by RT-qPCR using selected genes belonging to key altered pathways, such as cofactor biosynthesis (6-pyruvoyl tetrahydrobiopterin synthase), TCA cycle (type II citrate synthase), phenylalanine, tyrosine and tryptophan biosynthesis (anthranilate synthase component II), heme export (heme exporter protein D), mannose transport (PTS system mannose-specific transporter subunit IID) and nutrient transport (ABC transporter inner membrane subunit), with the latter also being a putative virulence factor (Table 1). Differential gene expression was confirmed with a significant correlation between RT-qPCR and RNA-seq (*r* = 0.95, *P* < 0.05).

**Table 1:** **Genes whose expression was validated using RT-qPCR with purified RNA from** ***H. parasuis*** **Nagasaki recovered after 2 h incubation in lung explants (ex vivo), 2 h after intratracheal infection (in vivo) and lung samples from a time-course intranasal infection (1 dpi, 2 dpi and 3 dpi)**

**Locus tag**	**Gene type**	**RT-qPCR** ^**1**^					ᅟ	**RNAseq**	
		**ex vivo**	**in vivo**	**1 dpi**	**2 dpi**	**3 dpi**	**Product**	**ex vivo**	**in vivo**
								**logFC**	**logFC**
HPNK_10441	Virulence factor	ND^2^	4.8	4.2	3.7	4.6	ABC transporter, permease protein	4.2	4.0
HPNK_06205	Virulence factor	ND	4.9	4.6	2.9	4.9	Hypothetical protein	2.1	6.0
HPNK_09829	Virulence factor	ND	3.6	4.1	1.4	3.3	Hypothetical protein	5.2	2.8
HPNK_10446	Virulence factor	ND	3.3	3.5	2.4	3.1	Hypothetical protein	3.1	5.5
HPNK_11461	Virulence factor	ND	5.2	2.1	2.2	2.9	Hypothetical protein	1.8	3.8
HPNK_06565	Virulence factor	ND	5.4	HC_T_^3^	HC_T_	3.7	Na+/H+ antiporter NhaC	2.3	3.3
HPNK_01698	Virulence factor	ND	4.1	3.8	3.2	3.0	VtaA2	1.6	3.1
HPNK_03728	Key pathway	2.3	5.0	ND	ND	ND	6-pyruvoyl tetrahydrobiopterin synthase	2.6	1.7
HPNK_08953	Key pathway	4.3	2.4	ND	ND	ND	ABC transporter inner membrane subunit	7.3	3.4
HPNK_06845	Key pathway	3.9	5.3	ND	ND	ND	Anthranilate synthase component II	9.1	1.8
HPNK_01064	Key pathway	1.3	5.9	ND	ND	ND	Heme exporter protein D	1.2	2.0
HPNK_10256	Key pathway	−2.8	0.6	ND	ND	ND	PTS mannose-specific transporter subunit	−3.2	−1.9
HPNK_05784	Key pathway	−4.2	−0.6	ND	ND	ND	Type II citrate synthase	−4.7	−3.4
HPNK_02274	Reference	ND	-	-	-	-	BirA repressor	−0.1	−0.1
HPNK_07913	Reference	ND	-	-	-	-	MurE ligase	−0.1	0.1
HPNK_00732	Reference	-	ND	ND	ND	ND	50S ribosomal protein L5	−0.5	−1.7
HPNK_07703	Reference	-	ND	ND	ND	ND	Translocation protein TolB	−0.2	−0.4
HPNK_09689	Reference	-	ND	ND	ND	ND	50S ribosomal protein L2	0.7	−0.6
HPNK_01728	Reference	-	ND	ND	ND	ND	pyruvate dehydrogenase subunit E1	0.0	−1.7

^1^log2 fold changes; ^2^Not determined; ^3^the gene was amplified but the high CT values obtained were not sufficient to calculate differential expression.

For the in vivo experiment, high-resolution Illumina sequencing was chosen. A total of 18 M and 15 M reads were obtained for the in vivo sample and the corresponding plate sample, respectively. From the total, 15% and 83% mapped to the Nagasaki genome, for in vivo and plate culture, respectively. This 15% accounted for 2.7 M reads that mapped to the Nagasaki genome from the total in vivo reads. To perform differential expression, a BCV of 0.3 was used, as obtained with the ex vivo data. Differential expression analysis between both samples yielded 542 DEG: 369 were up-regulated and 173 down-regulated after 2 h pig infection (Figure 1B and Additional file 2B). The differential expression was validated by RT-qPCR with the same genes used for the ex vivo experiment (Table 1). The correlation between the results by RT-qPCR and RNAseq was lower in this case but still statistically significant (*r* = 0.8, *P* < 0.05).

### Differential gene expression and enrichment

Gene set enrichments for housekeeping, up-regulated and down-regulated genes were performed in order to have an overview of *H. parasuis* transcriptomic response in the lung. With regards to the ex vivo incubation, analysis revealed 1430 genes whose expression remained constant with respect to their expression on agar plate. These stable genes were mainly related to intracellular processes, as evidenced by the enriched GO term “metabolic process”, encompassing the majority of the genes associated to biosynthetic processes. Among them, the most representative categories were amino sugar metabolism and other metabolic processes involved in amino acid, nucleic acid or ncRNA. Interestingly, some processes related to cell wall biogenesis were also constant, such as phospholipid, peptidoglycan and lipooligosaccharide biosynthetic processes, including lipid A metabolic process (Additional file 3A). Among the up-regulated genes in ex vivo conditions, GO enrichment revealed that genes related to “transporter activity”, within the “membrane” category, were enriched, as was also found in the in vivo experiment (see below). Some of these membrane-related genes were associated to pathogenesis functions (Additional file 4). Other ex vivo GO terms within the up-regulated genes were related to RNA processing, nucleic acid metabolic process, RNA metabolic process, including 21 genes related to tRNA processing (Additional file 3B). Among the most induced genes (*FDR* < 0.001) were: anthranilate synthase components I and II, glycerol-3-phosphate transporter, 2 hypothetical proteins, ABC transporter inner membrane subunit, anthranilate phosphoribosyltransferase, phage capsid scaffolding protein, and phage phi-c31 gp35-like protein. Notably, a high induction (>64 fold) of the operon related to anthranilate metabolism was observed after ex vivo incubation. Anthranilate is a key intermediate in aromatic amino acid biosynthesis and pyruvate production, and induction of its operon was also observed after in vivo infection (see below). On the contrary, the GO terms overrepresented among the set of down-regulated genes were related to intracellular pathways, with metabolism as the most represented, evidenced by the presence of GO terms related to stress response, sugar and ion transport, carbohydrate catabolism, tricarboxylic acid (TCA) cycle, nucleoside biosynthetic process, glyoxylate metabolic process and dicarboxylic acid metabolic process (Additional file 3C). Among the most repressed genes (*FDR* < 0.001) were the following: long-chain fatty acid transport protein P1/47 kDa outer membrane protein, methyl-galactoside ABC transporter galactose-binding periplasmic protein MglB, cold shock-like protein CspD, type II citrate synthase, universal stress protein A, maltose ABC transporter periplasmic protein, sigma 54 modulation protein and a hypothetical protein.

In an attempt to validate the ex vivo model, a short in vivo incubation was performed. Comparison of the DEG detected in both conditions showed considerable overlap; 120 genes were up-regulated in both conditions, belonging mainly to membrane–associated genes, mobile genetic elements and hypothetical proteins (Table 2 and Additional file 3D). In contrast to the stable genes, which were mainly related to intracellular metabolism, up-regulated genes after the in vivo infection consisted predominantly of the category “membrane”; with 57 genes that encoded membrane proteins, and 38 of those directly tagged as transporters. In addition, 14 of the up-regulated membrane genes were specific of the “outer membrane”, including several virulence associated trimeric autotransporters *vtaA*. Most of these genes were also up-regulated ex vivo (Additional file 4) and included functions usually related to virulence such as nutrient transport, drug export, adhesion and mobile genetic elements including transposases and phages. Some of the genes induced in both conditions were ABC transporter inner membrane subunit, ABC transporter permease protein, Na+/H+ antiporter, biopolymer transport ExbD protein, sn-glycerol-3-phosphate dehydrogenase subunit A, a drug/metabolite transporter (DMT) superfamily permease and several VtaA. In addition, the analysis also found genes that were induced only under one of the two conditions studied. Thus, genes uniquely induced ex vivo, although with subtle changes in vivo, included an iron-uptake permease inner membrane protein, an iron(III) ABC transporter ATP-binding protein, an oligopeptide ABC transporter permease and the peptide transport periplasmic protein SapA. However, some genes were only induced in vivo, but again with subtle changes ex vivo, such as the recombination protein 2, Mu-like phage gp25, heme/hemopexin utilization protein C/outer membrane receptor protein, Tfp pilus assembly pathway component PilC, competence protein E and phosphatidylglycerophosphatase B. Among the in vivo down-regulated genes, “metabolic process” was the most represented category with 125 genes, comprising both anabolic and catabolic processes. However, these genes were different from the ones found with stable expression. The most abundant metabolic categories were those related to translation, cellular carbohydrate metabolic process, TCA cycle, electron transport chain, nucleotide metabolic process, glycosyl compound biosynthetic process and monocarboxylic acid metabolic process (Additional file 3E). Forty-two genes in the “membrane” category were also found down-regulated, including carbohydrate and ion transporters. Key indicators of low growth rate during lung infection compared to plate culture were also revealed by the down-regulation of the ATP synthase subunits, responsible for generating ATP via the electrochemical gradient, as well as glucose and manose phosphoenolpyruvate-dependent sugar phosphotransferase systems (PTS). Also, 3 subunits of the Na(+)–NQR complex were down-regulated, suggesting anaerobic growth, as previously reported for other bacterial species [35]. The Na(+)–NQR complex plays an important role in bacterial energy metabolism [36].

**Table 2:** **Results of the comparison between the sets of differentially expressed genes (DEG) and enriched Gene Ontology (GO) terms between Nagasaki recovered after 2 h incubation in lung explants (ex vivo) or recovered 2 h after intratracheal infection (in vivo)**

**Gene group**	**Unique ex vivo**	**Shared**	**Unique in vivo**
DEGs UP	273	120	249
DEGs DOWN	273	99	74
GOs UP	74	5	46
GOs DOWN	68	115	102

**Figure 2:**
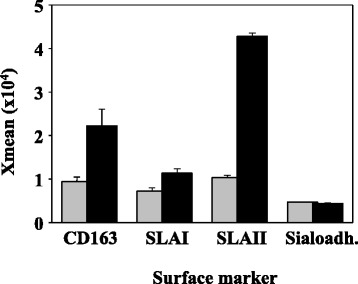
**Expression of surface CD163, SLAI, SLAII and sialoadhesin on alveolar macrophages.** The pig was intratracheally inoculated with *H. parasuis* strain Nagasaki (*black bars*). Infection was allowed to proceed during 2 h, when the animal was euthanized and alveolar macrophages were obtained from the lung by lavage. A control non-infected pig was included as the control (*gray bars*). Level of expression of the surface markers was measured with specific monoclonal antibodies by flow cytometry. Results (mean of the x-mean ± standard deviation) are representative of two independent analyses run with duplicate samples.

To support this model of short in vivo infection, alveolar macrophages from the BALF sample were analysed. Alveolar macrophages recovered after the 2 h infection, showed increased expression level of CD163, SLAI and SLAII when compared to the macrophages recovered from the non-infected pig. Sialoadhesin did not show different expression in the alveolar macrophages of the infected and the non-infected piglet (Figure 2). These results indicate that this short infection was enough for the animal to detect the presence of bacteria and respond to it.

### Up-regulated membrane genes and their conservation in virulent *H. parasuis* strains

The observation of numerous membrane-related genes that were up-regulated in both ex vivo and in vivo conditions was studied in more detail. Transmembrane topology and signal peptides were predicted in the putative proteome of Nagasaki using Phobius. A total of 225 putative proteins were predicted to have signal peptide, 360 to have transmembrane domains and 42 to have both signal peptide and transmembrane domains. These 627 genes are referred to as “membrane-related genes” for simplicity through the rest of the text. All these membrane-related genes in the Nagasaki genome were compared to the up-regulated genes in vivo or ex vivo, confirming the high proportion observed using GO terms. A total of 115 and 108 of those 627 were up-regulated ex-vivo and in-vivo, respectively. A comparison of these up-regulated membrane-related genes between ex vivo and in vivo confirmed the tendency observed with GO terms, with 36 shared genes. Next, in order to explore if these genes were unique to virulent strains, the list of up-regulated membrane-related genes was compared to a database of virulent and non-virulent genomic sequences of *H. parasuis*. We found 41 up-regulated membrane genes that were only present in virulent strains, or at most in one non-virulent strain. Twelve of these 41 genes were up-regulated in vivo and ex vivo, while 22 and 7 genes were uniquely up-regulated in vivo and ex vivo, respectively (Table 3). Shared up-regulated genes between the in vivo and ex vivo conditions included an ABC transporter permease, four genes of unknown function, Na+/H+ antiporter *nhaC*, a transposase mutator family protein and five virulence-associated autotransporters VtaAs (*vtaA*1, *vtaA*2, *vtaA*4, *vtaA*7 and *vtaA*11). The fact that *vtaAs* are assembled with no gaps in the Nagasaki genome used in the analysis strengthens the value of these results. The 22 genes uniquely up-regulated under in vivo conditions, included bacteriophage tail protein GPT, nine hypothetical proteins, three ISAs1 family transposases, putative iron compound ABC transporter permease, putative phage DNA replication protein O, two pyruvate kinases, two Sel1 domain repeat-containing proteins, and three *vtaAs* (*vtaA*3, *vtaA*8 and *vtaA*9). On the other hand, the 7 genes uniquely up-regulated in the ex vivo experiment included those encoding a CDP-diglyceride pyrophosphorylase, copper-transporting P-type ATPase, exonuclease III, two hypothetical proteins, phage-like minor tail protein and polysaccharide biosynthesis protein CapD (Table 3). Due to the essential role of pyruvate kinase enzyme in glycolysis, lack of this gene in non-virulent strains would be unlikely. The two genes annotated as pyruvate kinases and found only in virulent strains were HPNK_07393 and HPNK_09849. Further InterPro inspection results revealed that none of these two genes really belongs to the pyruvate kinase family (IPR001697), their protein annotation thus being incorrect. Although these two genes had a positive prediction for signal peptide, InterPro functional annotation was scarce. Their predicted domains are not specific to a particular pathway: a tetratricopeptide-like helical domain (IPR011990) for HPNK_07393 and a polyketide cyclase SnoaL-like domain (IPR009959) for HPNK_09849. In fact, there are at least three more genes annotated as “pyruvate kinase” in *H. parasuis* Nagasaki: HPNK_06225, HPNK_05314 and HPNK_07388. Only HPNK_06225 is specific to virulent strains, having a polyketide cyclase SnoaL-like domain (IPR009959). The other two, HPNK_05314, the largest one, seems to be the true pyruvate kinase (IPR001697), while HPNK_07388, again with a wrong annotation, has no predicted domains.

**Table 3:** **List of up-regulated membrane genes (** ***FDR*** **< 0.05) unique of virulent** ***H. parasuis*** **strains (V) or present at most in one non-virulent strain (one NV)**

**Locus**	**in vivo/ex vivo** ^**1**^	**Product**	**Unique V/one NV**
HPNK_10441	in vivo/ex vivo	ABC transporter, permease protein	Unique V
HPNK_03268	in vivo	Bacteriophage tail protein GPT	Unique V
HPNK_04602	ex vivo	CDP-diglyceride pyrophosphorylase	Unique V
HPNK_01144	ex vivo	Copper-transporting P-type ATPase	Unique V
HPNK_07008	ex vivo	Exonuclease III	Unique V
HPNK_00812	in vivo/ex vivo	Hypothetical protein	One NV
HPNK_06205	in vivo/ex vivo	Hypothetical protein	Unique V
HPNK_09829	in vivo/ex vivo	Hypothetical protein	Unique V
HPNK_10446	in vivo/ex vivo	Hypothetical protein	Unique V
HPNK_09189	ex vivo	Hypothetical protein	One NV
HPNK_00632	ex vivo	Hypothetical protein	Unique V
HPNK_00572	in vivo	Hypothetical protein	Unique V
HPNK_05179	in vivo	Hypothetical protein	Unique V
HPNK_00277	in vivo	Hypothetical protein	Unique V
HPNK_08083	in vivo	Hypothetical protein	One NV
HPNK_10351	in vivo	Hypothetical protein	Unique V
HPNK_00312	in vivo	Hypothetical protein	One NV
HPNK_11566	in vivo	Hypothetical protein	One NV
HPNK_11461	in vivo	Hypothetical protein	One NV
HPNK_04137	in vivo	Hypothetical protein	One NV
HPNK_04889	in vivo	ISAs1 family transposase	One NV
HPNK_00080	in vivo	ISAs1 family transposase	One NV
HPNK_04984	in vivo	ISAs1 family transposase	One NV
HPNK_06565	in vivo/ex vivo	Na+/H+ antiporter NhaC	Unique V
HPNK_11451	ex vivo	Phage-like minor tail protein	One NV
HPNK_07568	ex vivo	Polysaccharide biosynthesis protein CapD	Unique V
HPNK_05239	in vivo	putative iron compound ABC transporter permease	Unique V
HPNK_00090	in vivo	Putative phage DNA replication protein O	One NV
HPNK_09849	in vivo	Polyketide cyclase SnoaL-like domain protein	Unique V
HPNK_07393	in vivo	Tetratricopeptide-like helical domain protein	Unique V
HPNK_07408	in vivo	Sel1 domain protein, repeat-containing protein	Unique V
HPNK_07403	in vivo	Sel1 domain protein, repeat-containing protein	Unique V
HPNK_00210	in vivo/ex vivo	Transposase, Mutator family protein	One NV
HPNK_09439	in vivo/ex vivo	VtaA1	Unique V
HPNK_01479	in vivo/ex vivo	VtaA11	Unique V
HPNK_01698	in vivo/ex vivo	VtaA2	Unique V
HPNK_07258	in vivo	VtaA3	Unique V
HPNK_10146	in vivo/ex vivo	VtaA4	Unique V
HPNK_02582	in vivo/ex vivo	VtaA7	Unique V
HPNK_01967	in vivo	VtaA8	Unique V
HPNK_10812	in vivo	VtaA9	Unique V

^1^up-regulated in vivo, ex vivo or in both samples (in vivo/ex vivo).

Expression of four genes of unknown function, the *vtaA*2, the Na+/H+ antiporter *nhaC* and an ABC transporter permease protein was validated via RT-qPCR in the in vivo sample (Table 1). These genes were selected because they were found up-regulated in both in vivo and ex vivo conditions and were only detected in virulent strains. The up-regulation was confirmed for all seven genes, and positive correlation was found between RT-qPCR and RNA-seq (*r* = 0.8, *P* < 0.05). An increase in RT-qPCR fold change was correlated with an increase in RNA-seq fold change. In addition, the transcription of these genes was examined by RT-qPCR in BALF samples from a previous experimental infection with the Nagasaki strain. These genes were found to be up-regulated in bacteria recovered at 1, 2 and 3 dpi from the lungs of piglets intranasally inoculated with Nagasaki, with the exception of the Na+/H+ antiporter *nhaC*, whose expression was too low for evaluation by this method.

### ncRNA in *H. parasuis* and their differential expression

**Figure 3:**
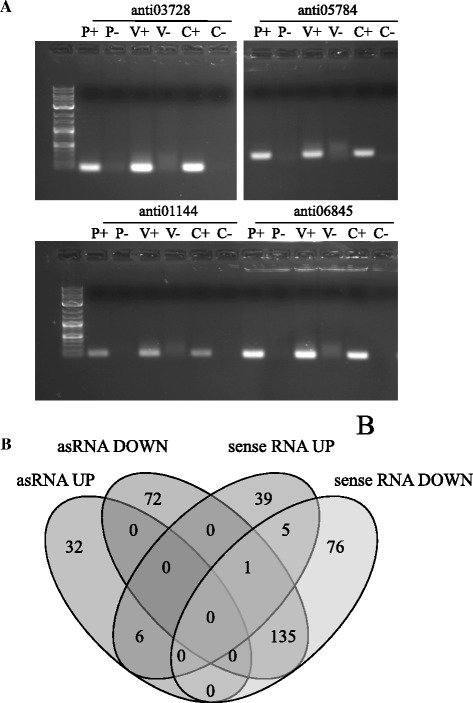
**Validation of antisense RNA (asRNA) expressed in vivo and in plate culture. A** Electrophoresis gel of four asRNA amplified by RT-PCR showing amplification results for RNA purified from plate culture with RT step (P+), plate culture without RT step (P-), 2 h in vivo sample with RT (V+) or without RT step (V-). Genomic DNA was included as the positive control (C+) and water as the negative control (C-). **B** Venn diagram showing overlapping Gene Ontology (GO) enriched terms for up- and down-regulated asRNA or sense RNA at 5% *FDR*.

Antisense expression was observed for all six sequenced samples, and was processed for further investigation. Count files from the counting step were used [GEO: GSE63851]. Determination of the sense/antisense expression ratio was performed. Due to the presence of some extreme values for antisense transcripts, median values were used. For ex vivo samples, sense to antisense ratios varied between 13 and 19, while a ratio of 43–68 was observed for their corresponding plate samples; thus evidencing a clear change in the sense to antisense ratio during ex vivo incubation. This tendency was also observed for the in vivo sample, where sense to antisense ratios of 4 and 15 were obtained for in vivo and its corresponding plate sample, respectively. These observations suggest that proportions were more equilibrated in the in vivo and ex vivo samples, while plate samples were biased towards sense expression. Normality of sense and antisense counts was assessed using the Shapiro-Wilk test. In all 6 cases *P* values were below 0.05 indicating non-normality. Therefore, correlations were assessed using the non-parametric Spearman rank-correlation test. The results indicate that in vivo sense and antisense expression was positively correlated (*r* = 0.43, *P* < 0.05). Similarly, plate sense and antisense expression was also positively correlated (*r* = 0.66, *P* < 0.05). However, this tendency was not observed for the ex vivo and their corresponding plate samples (*r* = 0.07-0.15, *P* < 0.05). All calculations were carried out using R statistical software [37]. Since a negative correlation would have been expected by reducing sense expression via sense/asRNA pairing, these unexpected results suggest that no sense/antisense regulation is occurring and that there are other factors influencing an increase in antisense expression. Pervasive transcription has been reported both for prokaryotes and mammals [38,39], but it has not yet been described for *H. parasuis*. To validate some of the observed asRNA, RT-PCR was performed. Four asRNA were validated both in the in vivo and plate culture samples. These asRNA overlapped with genes annotated as 6-pyruvoyl tetrahydrobiopterin synthase, type II citrate synthase, copper-transporting P-type ATPase and anthranilate synthase component II (Figure 3A and Additional file 1). The positive correlation sense-asRNA observed for the in vivo sample was studied in more detail. Antisense differential expression was calculated, finding a considerable number of differentially expressed asRNA (*FDR* < 0.05), 213 and 268 up- and down-regulated, respectively (Additional file 2C). GO enrichments were also performed for the set of differentially expressed asRNA (Additional files 3F and G) and were compared to sense enrichment results. We found an important overlap between the enriched GO terms of the repressed asRNA and sense RNA, with 135 matches (Figure 3B). These pathways were mainly related to intracellular genes, mostly metabolism. Among them, carbohydrate catabolic process, phosphoenolpyruvate-dependent sugar phosphotransferase system, translation, tricarboxylic acid cycle, glycosyl compound biosynthetic process, purine ribonucleoside triphosphate biosynthetic process and monocarboxylic acid metabolic process were the main representatives. These results confirm that the positive correlation of sense and antisense expression was also linked to some common pathways. Some overlap was also observed between the pathways of the induced asRNA and sense RNA, with 6 shared GO terms including pathogenesis and pilus organization.

## Discussion

After an initial colonization of the upper respiratory tract, lung infection is one of the early steps in *H. parasuis* pathogenesis. Therefore, a deep understanding of the factors involved in survival of this bacterium in the lung will be useful for disease control. In this study, we have shown that the Nagasaki strain of *H. parasuis* modulates its gene expression during in vivo lung infection, but also in an ex vivo lung infection model. Nutrient acquisition, expression of genes related to pathogenesis and a reduced metabolism are the main signatures of *H. parasuis* adaptation to the lung environment. Some of the up-regulated genes during lung infection were only found in the genomes of virulent strains, indicating their potential as virulence factors. In addition, previously reported *H. parasuis* virulence factors were also up-regulated in the lung.

One of the most important limitations to elucidate bacterial gene expression during host colonisation is the low RNA quality that is usually obtained from field or experimental infections. Moreover, the proportionally low quantity of mRNA relative to rRNA makes transcriptomics a challenging strategy. In our case, we were not able to obtain enough RNA of good quality for high-throughput sequencing from samples taken at 1 to 4 days post-inoculation from an infection previously performed by us [14,27]. To overcome these limitations, we decided to use a short in vivo infection and an ex vivo lung infection model. Processing of BALF samples for RNA purification coupled with additional enrichment steps such as differential centrifugation to remove host cells and removal of host and bacterial rRNA resulted in a modest but sufficient RNA quantity to study bacterial gene expression by metatranscriptomics. In our study, 3 M reads from the ex vivo sample and 18 M reads from the in vivo sample were enough to study the gene expression of 95% of the genes. These sequencing depths were sufficient to reach the coverage recommended to quantify bacterial gene expression [40].

In vivo incubation revealed numerous genes that were differentially expressed. Even prior to pathway analysis, visualization of the MA differential expression plot showed a particular “wave” shape, thus pointing to up-regulation of genes normally not expressed during laboratory agar plate growth, and, conversely, down-regulation of the most expressed ones. Increased nutrient capture, together with metabolism repression were the main adaptive responses of *H. parasuis* in the lung. Interestingly, a similar picture has been observed in other in vivo transcriptome studies of other bacterial species from the *Pasteurellaceae* family, such as *Mannheimia hemolytica*, responsible for bovine pneumonic pasteurellosis [17] or for *Actinobacillus pleuropneumoniae*, the etiological agent of porcine pleuropneumonia [18], thus suggesting slower metabolism and replication rate in vivo compared to growth in a rich culture media. Deslandes et al. [18] also found that the "Transport and Binding Protein" functional class was overrepresented among the up-regulated genes in *A. pleuropneumoniae* under in vivo conditions. Here, we found that the most represented cellular component among the up-regulated genes in the lung was the one related to membrane proteins. Some of these genes were distributed in different categories of nutrient capture, such as sugar and amino acid transport. These findings indicate that “nutritional virulence”, previously reported for other bacterial pathogens [41], could be an important survival strategy for *H. parasuis* during lung colonization. Moreover, some of these genes were involved in iron, nitrate and sulfonate acquisition, which are vital for energy generation and have been previously reported to be up-regulated in *H. parasuis* grown under iron-restriction in vitro [42]. Furthermore, up-regulation of cytochrome biogenesis, heme transport and some components of the electron transport chain add additional value to the hypothesis that maintenance of anabolic/catabolic balance could be important for survival in the host. Additionally, ABC transporters, which have been found to be up-regulated in the current study, have been reported to be essential elements for bacterial survival in the host [43,44]. Interestingly, some ABC transporters could have been acquired via horizontal gene transfer in *H. parasuis* [23]. While ABC transporters were found up-regulated, the PTS sugar transport systems were down-regulated, indicating that different sugar transporters could be used depending on the environmental conditions, by opposite regulation of these two ATP-dependent transporters [45].

One of the main questions we tried to answer in this work was if any of the previously reported virulence factors were up-regulated in vivo. Genes involved in surface polysaccharides, lipid A or proteases did not show an overall increased expression. However, iron, hemin, hemopexin or transferring binding proteins showed a tendency for up-regulation. Also, mining for up-regulated membrane-related genes that were unique to virulent strains revealed 42 putative virulence factors. Several of these gene categories have been previously linked to virulence in bacteria, such as iron acquisition or ABC transporters [46], frequently subjected to horizontal genetic transfer via mobile genetic elements [47]. On the contrary, some proteins previously reported as putative virulence factors in *H. parasuis*, such as outer membrane protein P2 (OmpP2), cytolethal distending toxins, heptosyltransferases, which have been involved in adhesion, invasion or serum resistance [48-50], showed subtle down-regulation. This down-regulation could indicate that these molecules do not play a specific role in lung survival. Finally, *vtas* were up-regulated in vivo, including *vtaA8* and *vtaA9*, which have been demonstrated to play a role in phagocytosis resistance in *H. parasuis* [15]. Due to the complex structure of these genes, with repetitions, common domains and variability of the passenger domain [51], careful analysis should be performed to determine their presence in different strains. Previous knowledge about these genes indicates that *vtaA*1 to *vtaA*11 are associated with virulence [52]. We should highlight that a total of 128 up-regulated genes are currently annotated as genes of unknown function. Some of these genes of unknown function were detected by Phobius as membrane-related and were only found in virulent strains. These genes of unknown function require further research, since they could play a role in novel pathogenic strategies [53]. Additionally, the validation of up-regulation of 4 of these genes of unknown function in other in vivo samples supports the role of these membrane proteins during infection as well as their use as vaccine candidates.

Development of ex vivo models has been reported as useful to mimic in vivo conditions [54]. Currently, an ex vivo infection model is absent for *H. parasuis*. In this work we tried to bypass the current limitations of in vivo experiments by developing an ex vivo lung infection model. A 2 h ex vivo incubation resulted in high yield of bacterial RNA recovery. Importantly, DEG were detected in similar pathways as in the in vivo sample, validating this strategy as a good alternative for *H. parasuis* host-pathogen interaction studies at the respiratory tract. However, the comparison of the results from the ex vivo and in vivo infections also found differences between these samples, indicating that the true expression of some genes during infection can only be revealed in vivo. The advent of ultra-high throughput sequencing would allow previously unimagined sequencing depths, which will be useful in the analysis of samples from in vivo experiments. For instance, analysis of other sample types containing a lower number of bacterial cells, such as samples from later stages of infection, will open new possibilities to study the progression of disease in the lung.

One of the key factors to develop an effective vaccine to control a bacterial infection is to know its pathogenic lifestyle. We found that *H. parasuis* changes its global gene expression during lung infection, with an overall tendency to up-regulate membrane-related genes involved in carbon acquisition, iron binding and pathogenesis. A strong down-regulation of metabolism was observed, however, suggesting an importance of a lower replicative rate as an adaptation for host survival. These metabolic adaptations were accompanied by induction of well-known virulence factors as well as other genes that, although less explored, could be behind novel virulence mechanisms. In summary, this work serves as a useful infection model for *H. parasuis,* adding value to some up-regulated virulence factors for their use as vaccine candidates.

## Additional files

Additional file 1:
**Primers.** Primers used to validate expression pattern of RNA-seq data. Additional file 2:
**Differential expression of Nagasaki genes.** Differential expression of genes ex vivo versus plate culture (A), in vivo versus plate culture (B) and antisense RNA in vivo versus plate culture (C). Additional file 3:
**GO enrichments.** Gene set enrichments for housekeeping, up-regulated and down-regulated genes. Enriched GO categories among the up- and down-regulated antisense RNA in vivo are also shown. Additional file 4:
**Membrane genes.** Membrane genes up-regulated in vivo, ex vivo or in both conditions. Differential expression is also shown.
